# Higher anaerobic digester performance by the strategical increase in the feeding rate of cow manure in laboratory continuous stirred tank reactor

**DOI:** 10.1371/journal.pone.0332972

**Published:** 2025-10-17

**Authors:** Vahed Negahban, Mohammadali Ebrahimi-Nik, Abbas Rohani

**Affiliations:** Department of Biosystems Engineering, Faculty of Agriculture, Ferdowsi University of Mashhad, Mashhad, Iran; Tsinghua University, CHINA

## Abstract

Improper management of dairy cow manure poses a major risk of environmental pollution. In contrast, anaerobic digestion provides a sustainable pathway by transforming manure into renewable biogas while reducing environmental burdens compared with conventional handling methods. This study examined the operational adaptation and performance of a continuous stirred-tank anaerobic digester fed with dairy cow manure, where the daily organic loading rate (kgVS/m³ reactor volume) was gradually increased to maximize biogas generation. The loading was progressively raised until clear signs of process instability appeared. The experiment was conducted over 98 days under mesophilic conditions, with pH maintained between 7.10 and 7.40. Initially, the digesters were supplied with 20 g of fresh manure per day (0.49 gVS/L), and the input was doubled weekly until reaching 280 g/day (6.82 gVS/L). Under optimal conditions, peak biogas and methane productions were 16.2 NL and 9.2 NL, respectively, corresponding to a maximum specific methane yield of 232 NL CH₄/gVS. The results show that higher loading rates stimulated microbial activity and accelerated organic matter degradation, leading to substantially greater biogas output. Moreover, the digestate exhibited improved physicochemical characteristics, enhancing its value as a nutrient-rich amendment for agricultural applications.

## 1. Introduction

Technological progress and population growth are closely interlinked, with each reinforcing the other [1]. With global population projections anticipated to reach 9–10 billion by 2040, the demand for energy and raw materials is expected to increase substantially [2]. This rising demand presents a dual challenge: decreasing dependence on fossil fuels while minimizing the environmental consequences of their consumption [3]. Fossil fuel combustion remains a major contributor to anthropogenic CO₂ emissions, which are recognized as a key driver of global warming. Furthermore, international initiatives, such as the 2015 Paris Agreement, have emphasized the urgent need to diversify energy portfolios and adopt carbon-neutral alternatives like bioenergy to mitigate climate change and promote sustainable development [4].

Biogas represents a renewable energy source that can serve as a viable substitute for fossil fuels in both heat and electricity generation [5]. In this context, anaerobic digestion (AD)—an efficient and environmentally friendly technology—has attracted substantial attention [6], with its adoption steadily increasing over the past decade [7]. At present, AD accounts for roughly 10% of the global primary energy supply, and biofuel production is expected to increase by 25% by 2024, underscoring the expanding role of AD in meeting global energy demands while mitigating associated environmental challenges [8]. The performance and stability of anaerobic digesters are critically influenced by the characteristics and origins of the organic feedstocks used. These feedstocks can be broadly classified into five categories: (i) sewage sludge (SS); (ii) animal manures; (iii) food industry wastes, including by-products from slaughterhouses; (iv) energy crops and harvesting residues, such as algae; and (v) the organic fraction of municipal solid waste (OFMSW) [9].

In recent decades, the global expansion of the human population has been accompanied by a marked rise in dairy farming. Current estimates suggest that the global cattle population exceeds 1.5 billion, producing nearly 40 million metric tons of waste annually, a large proportion of which is manure [10]. Dairy farms generally generate both liquid and semi-liquid manure, with volumes varying according to the amount of freshwater used in daily operations [11]. Intensive farming practices in many Asian and European countries further contribute to this waste burden. For example, livestock operations in Europe alone generate more than 1.4 billion tons of organic waste—including manure—each year [12]. Each cow produces approximately 30 kg of manure per day, creating significant management challenges for the cattle industry [13]. The manure is nutrient-rich, containing high levels of minerals, carbon, nitrogen, heavy metals, and diverse microbial communities [14]. While in many regions cattle manure is applied as a bio-fertilizer to improve crop yields, its improper use can result in nutrient and heavy metal accumulation, soil degradation, and broader environmental contamination [15]. Moreover, the release of cattle manure into the environment can cause offensive odours, water pollution, and the spread of pathogenic bacteria, thereby threatening public health. The decomposition of 1 kg of cow dung releases an estimated 312 kg CO₂ equivalent of greenhouse gases, further intensifying the global climate crisis [13]. Consequently, the implementation of effective mitigation strategies is imperative to prevent the pollution associated with cattle manure [10].

Biogas production from animal manure has attracted increasing attention as an innovative strategy for transforming waste into renewable energy. By employing advanced technologies, the environmental impacts associated with manure disposal can be substantially mitigated, while its value as a clean energy resource is simultaneously enhanced. Anaerobic microbial consortia have shown significant potential for efficiently converting animal manure into biogas [16]. The anaerobic digestion process is carried out by a diverse consortium of microorganisms across four key stages. During the hydrolysis stage, complex organic matter and microbial cells are degraded into soluble compounds. In the subsequent acidogenesis stage, these compounds are converted into volatile fatty acids (VFAs) by acid-producing bacteria. During acetogenesis, VFAs are further metabolized, yielding primarily acetic acid; together with acidogenesis, these steps are commonly referred to as fermentation. Finally, in the methanogenesis stage, methanogenic archaea convert VFAs and hydrogen into methane (biogas) and carbon dioxide. Disruption at any of these stages can adversely affect the process, potentially causing instability in biogas production. Frequent operational challenges—such as organic overload, hydraulic overload, and ammonia inhibition—underscore the necessity of continuous monitoring to ensure system stability [17]. In addition to biogas, anaerobic digestion (AD) yields a byproduct known as digestate—a stabilized residue of degraded organic matter [18]. Digestate serves as a nutrient-rich soil amendment and organic fertilizer, reducing dependence on synthetic fertilizers while improving soil health and crop productivity [19]. Its application may even replace synthetic fertilizers, such as urea, thereby promoting sustainable agricultural practices [13]. Consequently, digestate utilization not only provides environmental benefits but is also economically advantageous. Furthermore, the commercialization of processed digestate offers an additional revenue stream for biogas plant operators, enhancing both energy recovery and overall sustainability [10].

The anaerobic digestion (AD) process is a complex and interdependent biological system, in which factors such as substrate characteristics, temperature, buffering capacity, and microbial activity play critical roles. At each stage, these components must satisfy specific requirements to ensure optimal system performance. Inadequate control or adjustment may lead to process imbalances, reduced substrate degradation, impaired digestate quality, and ultimately lower biogas yields [20]. Among the operational parameters, the organic loading rate (OLR) is particularly important for maintaining digester performance and stability. Excessive OLR values can lead to the accumulation of volatile fatty acids (VFAs), thereby disrupting the digestion process [21]. Higher OLRs typically enhance bacterial activity during the hydrolysis and acidogenesis stages but divert metabolic balance away from methanogenesis, resulting in the buildup of VFAs [22]. VFAs, short-chain organic acids (C2–C6) produced by acidogenic bacteria, serve as critical intermediates in the anaerobic fermentation of complex organic substrates [23]. Their concentration within a digester is a key diagnostic indicator of process health and efficiency. The primary VFAs—acetic, propionic, and butyric acids—often accumulate when the symbiotic balance between acidogenic and methanogenic microorganisms is disturbed. Such imbalance may lower pH, which in turn accelerates further VFA accumulation. Additionally, limited nutrient availability can elevate acetate and hydrogen production, and under high hydrogen partial pressures, this condition promotes the accumulation of propionic and butyric acids [24]. Effective mitigation of VFA accumulation is therefore essential for operating digesters at higher OLRs and shorter hydraulic retention times (HRT), thereby improving digestion efficiency and maximizing biogas production [25]. The optimal OLR is reached when methane yield per unit volume of the digester is maximized while maintaining process stability, as methane productivity increases linearly with rising OLR until the system becomes overloaded [26]. Accordingly, this study seeks to determine the maximum sustainable daily feeding rate (kg VS per m³ digester) by incrementally increasing the feed rate and assessing its potential to enhance biogas yield during the continuous anaerobic digestion of dairy cow manure.

## 2. Materials and methods

### 2.1. Biogas digester structure

**Fig 1:**
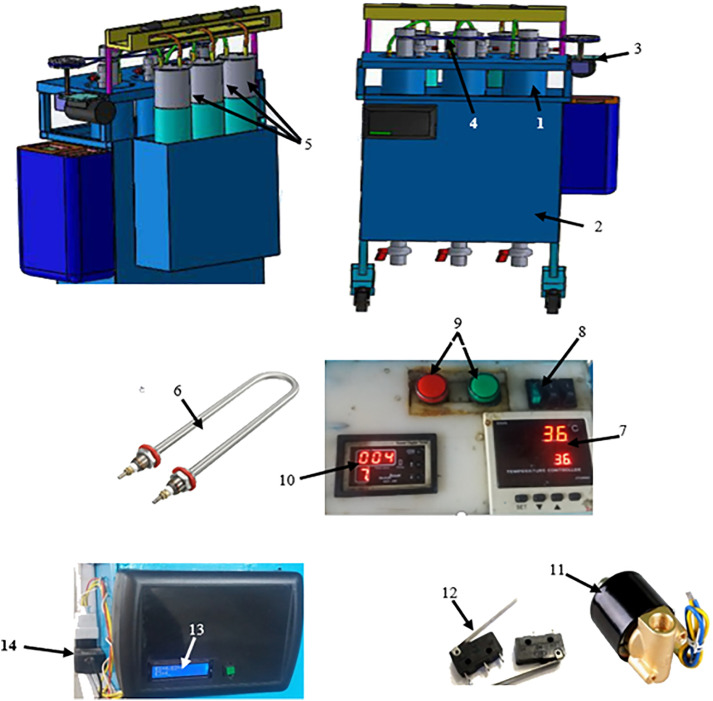
Overview of the anaerobic digestion system: 1. Biogas digester, 2. Oil box, 3. Electric motor, 4. Belt mechanism, 5. Biogas storage tanks, 6. U-shaped heating element, 7. Relay switch, 8. Connection switch, 9. Active on/off switch, 10. Digital timer, 11. Geert brand solenoid valve, 12. Lever micro-switch (OMRON 30 mm model), 13. Biogas discharge counter, 14. Power adapter.

The biogas reactor system used in this study consisted of three independent digesters. Each digester was equipped with a gas outlet valve, material inlet and outlet valves, an electric motor, a mechanical stirrer, and various ancillary components, including hoses and a solenoid valve, as well as a cylindrical gas storage tank. Each digester had an effective volume of 6 L and was positioned 8 cm apart within a square frame measuring 54 cm in both length and height, with a wall thickness of 3 mm. The upper section of each digester contained two valves—one for substrate entry and one for gas release—along with bearings and an electric motor that drove the stirring blades via a zigzag belt. Uniform mixing was achieved by rotating the stirring axis and attached blades at 110 rpm for 1 minute, followed by an 8-minute rest period (Fig 1)

Temperature control in each digester was maintained using heating elements immersed in burnt oil under mesophilic conditions. Burnt oil was selected for its corrosion resistance, low evaporation rate, and high thermal conductivity, with both the heating elements and the temperature sensor fully submerged in the oil. Temperature is a critical factor in anaerobic digestion, particularly during the methanogenic phase, as elevated temperatures accelerate substrate degradation and biogas production [27].

Each digester was connected to a gas storage tank designed to capture and retain the biogas produced. These tanks, constructed from polycarbonate, have a volume of 4.5 L (10.5 cm in diameter and 50 cm in height) and are enclosed within a secondary polycarbonate cylinder with an effective volume of 6.8 L (12 cm in diameter and 60 cm in height). To facilitate long-term gas storage, a novel water displacement method was employed: as biogas enters the tank, it exerts pressure on the water surface, causing the tank to float upward. This movement generates a height difference between the two cylinders, and by measuring the volume of displaced water, the volume of biogas produced can be accurately determined using the following equation:


$$V_{d}=\left(\pi r^{\text{2}}\;\Delta H\right)\times T_{n}$$ (1)

Where, V_d_ is the effective volume of biogas produced (mL), r is the radius of the displaced cylinder (cm), ΔH is the height difference between the two biogas cylinders (cm), Tn is the temperature and pressure correction factor to standardize the volume of biogas produced.

### 2.2. Raw materials for testing

#### 2.2.1. Dairy cow manure.

The primary substrate for this study was dairy cow manure, sourced from the cattle farm at Ferdowsi University of Mashhad (Mashhad, Iran). The manure was collected in separate containers and transported to the biogas laboratory for analysis and experimental use. After collection, it was stored at 4°C until required for the experiments [28]. Typically, manure was collected every 10 days over a six-month period to ensure a consistent supply

#### 2.2.2. InoculumFresh dairy cow manure from the same farm was also used as the inoculum.

The manure was diluted with water in a 1:1 ratio, and a measured quantity of this mixture was added to each digester. The inoculum was maintained in the digesters for two months without additional feed or additives, allowing the microbial population to establish and stabilize. During this acclimation period, the bacterial feed contribution gradually decreased until it reached zero, ensuring that the microbial community was fully adapted before the experimental feeding regime commenced.

### 2.3. Test method

The biogas digester system comprised three independent digesters housed in an oil-filled enclosure to maintain a stable temperature. One digester served as the control, while the remaining two were operated as replicates. Daily feeding and biogas measurements were performed at a fixed time using a dedicated container for feeding and a scale for mass determination. The collected cow manure was weighed and mixed with water in a 1:1 ratio before being added to each digester. The feed quantity was calculated using Eq. (2).

During the first week, each digester received 20 g of fresh manure per day. In subsequent weeks, the feed was incrementally increased by 20 g per week until signs of process instability were observed. This stepwise approach allowed the determination of the optimal feeding level for maximum biogas production. The feeding procedure required temporarily removing the digesters. The daily feed amount was determined according to Eq. (2) from [29]. Each digester had an effective volume of 5.25 L, and the manure contained 16% total solids (TS) and 80% volatile solids (VS). The organic matter content of the feed was calculated as 0.5 kg VS/m³ [29]. Considering that 1 m³ = 1000 L and 1 kg = 1000 g, the feeding amount was expressed in kg/day using Eq. (2):


$$m(kg/d)=\frac{B_{R}\times V_{R}}{C}$$ (2)

Where, m represents the feeding amount in kg/day, V_R_ refers to the digester effective volume in liters, C indicates the concentration of organic matter as a percentage of total solids (TS) and volatile solids (VS), and B_R_ is the organic matter concentration in kgVS/m³ d.

To calculate the feeding amount based on VS, several factors were considered, including digester feed rate, TS and VS content of the manure, and the digester effective volume. Eq. (3) was applied to determine the amount of VS in each digester:


$$P_{CH\text{4}}=\frac{V\;\times C}{V_{R}}$$ (3)

Where, P_CH4_ represents methane production per feed VS/L in gVS/L, V_R_ is the effective digester volume in liters, V is the digester feed volume in L/d, and C is the concentration of organic matter as a percentage of TS and VS.

#### 2.3.1. Total solids percentage (%TS).

**Fig 2:**
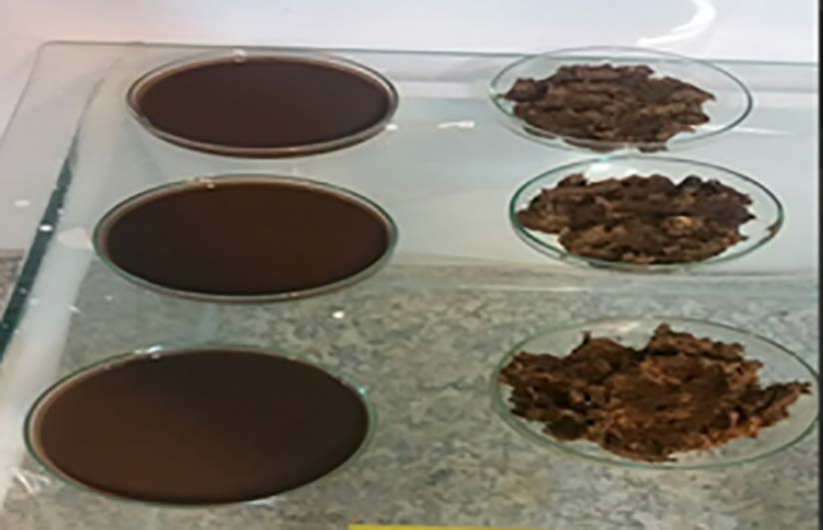
Fertilizer and inoculum samples dried at room temperature for 24 hours.


The percentage of total solids (TS) in the samples was determined using the following procedure. First, a Petri dish was preheated in an oven at 105°C for 1 hour. After preheating, the dish was cooled in a desiccator to prevent moisture absorption. A 50 g sample of each material was then placed in three separately pre-weighed Petri dishes. The samples were left at room temperature for 24 hours to allow initial moisture evaporation (Fig 2). Subsequently, the Petri dishes containing the samples were dried in an oven at 105°C for 24 hours. After drying, the dishes were cooled again in the desiccator to avoid moisture uptake. The weight of each Petri dish was measured after cooling. If the weight difference between two consecutive measurements exceeded 4%, the drying process was repeated for an additional hour. This procedure was continued until the weight difference between consecutive measurements was below 4%, ensuring complete removal of moisture. The percentage of total solids (%TS) was calculated using Eq. (4) [28]:

$$TS=\frac{\left(A-B\right)}{\left(C-B\right)}\times \text{1}\text{0}\text{0}$$ (4)

Where A is the weight of the dried sample and Petri dish in grams, B is the weight of the empty Petri dish in grams, and C is the weight of the sample and Petri dish before drying in grams.

#### 2.3.2. Volatile Solid percentage (VS).

The volatile solids (VS) percentage of each sample, based on its dry weight, was determined using the combustion method in an electric furnace. The required equipment included a laboratory balance, electric furnace, desiccator, porcelain crucible, and crucible clamp. The procedure began with homogenizing the samples, ensuring they were fully powdered to facilitate accurate total solids measurement. Subsequently, 2 g of each sample was weighed into a pre-weighed porcelain crucible. The crucibles were then heated in the electric furnace at 550°C for 1 hour, followed by cooling in a desiccator and weighing. This heating–cooling cycle was repeated for an additional 30 minutes until the weight difference between two consecutive measurements was less than 4%, indicating complete removal of volatile solids. The volatile solids percentage (VS) was calculated using Eq. (5) [28]:


$$VS=\frac{\left(A-D\right)}{\left(A-B\right)}\times \text{1}\text{0}\text{0}$$ (5)

where A is the weight of the initial dried sample plus crucible (g), B is the weight of the empty crucible (g), and D is the weight of the sample plus crucible after heating (g).

#### 2.3.3. Biogas volume measurement.

Each digester was equipped with an individual 4.5 L gas storage tank where biogas accumulated on a daily basis. The gas was discharged through an electric valve actuated at predetermined micro-switch heights. Prior to feeding, the digesters were stirred for 1 minute using an electric stirrer controlled by a timer. This stirring step prevents solids from settling at the bottom of the digester and ensures uniform distribution of organic matter, thereby facilitating optimal nutrient availability for the microbial community. After each feeding, the gas counter was reset to zero to enable accurate measurement of the subsequent gas volume. To quantify biogas production and allow comparison with other studies, the normalization coefficient given in Eq. (6) was applied. This equation accounts for the initial temperature (T₁), volume (V₁), and pressure (P₁) of the gas, as well as the temperature (T₂), volume (V₂), and pressure (P₂) under standard conditions. Standard conditions are defined as 1 atm pressure and 0°C (273.15 K).


$$\frac{\text{P1 V1}}{\text{T1}}=\frac{\text{P2 V2}}{\text{T2}}$$ (6)

#### 2. 3. 4. Methane production measurement in biogas.

**Fig 3:**
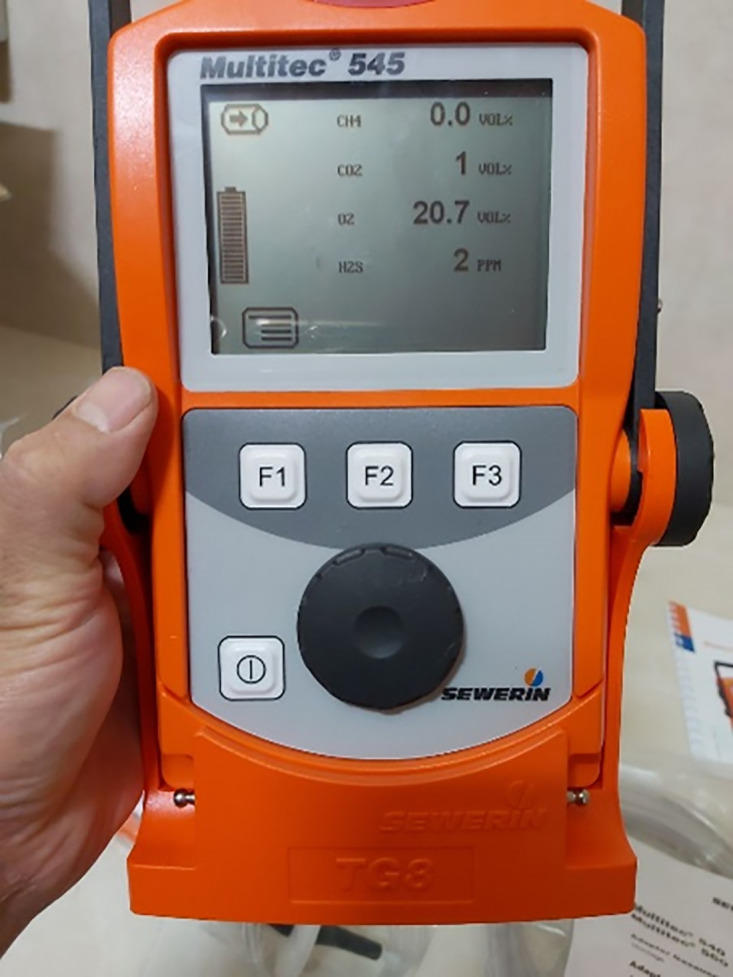
MultiTech 545 gas analyzer used for biogas composition analysis.

A MultiTech 545 gas analyzer was employed to determine the concentrations of CH₄, O₂, and H₂S in the produced biogas. The measurement system consisted of a solenoid valve and a connecting hose linking the digester to the analytical device. Prior to analysis, the biogas was filtered and then introduced directly into the analyzer through the connecting hose (Fig 3).

### 2.4.. Statistical analysis of biogas production and related parameters

To evaluate the temporal trends and stability of biogas production during anaerobic digestion, a repeated measures analysis of variance (RM-ANOVA) was performed [30]. This approach is particularly suitable for experimental designs involving multiple measurements over time on the same experimental units. In the present study, biogas production was monitored in triplicate on each sampling day over a 100-day period. The variables analyzed included methane yield, total biogas volume, pH, and methane concentration (%CH₄), with time (sampling day) defined as the repeated factor.

The statistical analysis was carried out using MATLAB’s *fitrm* and *ranova* functions, which account for within-subject correlations and enhance statistical power by minimizing inter-subject variability. Assumptions of sphericity were evaluated, and when violations were detected, appropriate corrections (Greenhouse–Geisser, Huynh–Feldt, and lower-bound adjustments) were applied to ensure valid inference.

In addition to hypothesis testing, 95% confidence intervals were calculated and plotted for each response variable, including cumulative biogas production, methane yield, methane concentration, and pH. These confidence bands facilitated the interpretation of variability patterns and provided a robust assessment of the overall stability and consistency of the anaerobic digestion process under the experimental conditions

## 3. Results and discussion

The physicochemical properties of dairy fresh cow manure, inoculum, and digestate are summarized in Table 1. Key parameters, including total solids (TS), volatile solids (VS), and nutrient composition, provide a baseline for evaluating the efficiency of the anaerobic digestion process. The differences observed between the fresh feedstock and the resulting digestate reflect the transformation of organic matter and nutrients during digestion, thereby establishing a foundation for the subsequent discussion on biogas production and methane yield

**Table 1:** Physicochemical properties of dairy fresh cow manure, digestate, and inoculum resulting from anaerobic digestion.

Parameter	Unit	Digestate	Inoculum	Dairy Fresh Cow Manure
Total Solids (TS)	%	5.07	5.49	16.18
Volatile Solids (VS)	%TS	78.83	73.5	80.83
Carbon (C)	%TS	–	24.19	35
Nitrogen (N)	%TS	1.6	1.05	1.54
Phosphorus (P)	%	0.487	0.32	0.446
Potassium (K)	%	1.11	1.02	0.7

### 3.1 Total Solid (TS) Content

The composition of cow manure is influenced by the diet and digestive processes of individual cows, leading to variations in carbohydrate, lipid, and protein content. These differences primarily reflect the daily dietary requirements of each cow type. Manure characteristics also vary depending on the feedlot conditions, feeding and digestion processes, and feeding schedules of the animals [31] In this study, the total solids (TS) content was measured at 16.18% for dairy fresh cow manure and 5.49% for the inoculum used as an activity starter (Table 1). These values are consistent with literature reports, where cow manure typically exhibits TS values between 14% and 25%, and inoculum TS ranges from 6% to 7.2%, depending on the system and operational conditions employed. For instance, Wang et al. (2019) reported TS contents of 14.6% for cow manure and 7.16% for inoculum in a laboratory-scale digester [32], whereas Miah et al. (2016) observed 17% TS for manure and 6% TS for inoculum under similar experimental conditions [33]. Higher TS values, such as 24.43% for cow manure, have also been reported by Chen et al. (2010) in small-scale bottle digesters, indicating variability influenced by feedstock characteristics and pre-treatment methods [34]. The TS values obtained in this study fall within the expected range, demonstrating consistency with established benchmarks. This validates the feedstock and inoculum preparation methods used and provides a reliable foundation for evaluating subsequent digestion performance and biogas production.

### 3.2. The Ratio of carbon to nitrogen (C/N)

The carbon-to-nitrogen (C/N) ratio is a critical determinant of anaerobic digestion efficiency, as it strongly influences microbial metabolism and process stability [35]. Maintaining a balanced C/N ratio ensures an adequate nutrient supply for microbial activity while minimizing ammonia accumulation, which can inhibit digestion performance [36]. Optimal anaerobic digestion generally occurs within a C/N ratio range of 20–30 [37], although ratios between 13.9 and 19.6 have also been reported as effective under certain conditions [38]. Variations in C/N ratios may result from cattle breed, environmental factors, and feed composition, all of which affect manure characteristics. In this study, the C/N ratio of cow manure was approximately 22.7, while the inoculum exhibited a slightly higher ratio of 23. These values fall within the optimal range, indicating that both substrates were well-suited for microbial activity. Consistent with these results, Abdelsalam et al. (2016) reported C/N ratios of 26:1 for cow manure and 24:1 for slurry, further confirming the suitability of these substrates for initiating and sustaining anaerobic digestion [39]. Overall, the findings underscore the appropriateness of the selected feedstock and inoculum for achieving stable and efficient digestion.

### 3.3. Influence of acidity (pH) on methane production

**Fig 4:**
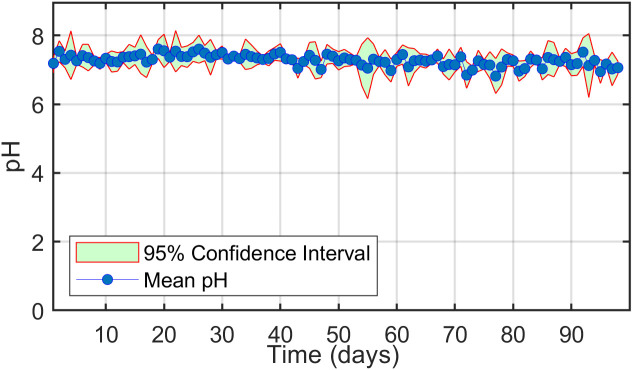
Monitoring of pH dynamics over time in the anaerobic digestion system.

pH is a critical parameter in anaerobic digestion, directly influencing methanogenesis and methane yield. To ensure optimal conditions, the pH of the digestate was measured daily throughout the experiment, accounting for the time-intensive setup of the digestion system and the continuous feeding process (Fig 4). The ideal pH range for methanogenesis in inoculum has been reported to be 7.10–7.40 [40]. Deviations from this range can impair bacterial activity, leading to reduced methane production [41]. In this study, the digestate pH remained within the optimal range of 7.30–7.40 during the first 42 days. A gradual decline in pH was observed between the 42nd and 44th days, coinciding with an increased feeding rate and a drop in laboratory temperature. As the temperature stabilized and the microbial community adapted to the feeding changes, the pH returned to its optimal range. A more pronounced pH decline occurred between the 71st and 74th days, falling below 7.10 due to a sharp temperature drop coupled with increased feeding, highlighting the sensitivity of microbial activity to environmental changes. Subsequently, as the temperature rose and bacteria acclimated, their activity recovered, and pH stabilized at 7.15–7.30. From day 92 onward, another increase in feeding caused a slight pH decrease, reaching 7.10–7.18.

These fluctuations underscore the critical interplay between feeding rate, environmental temperature, and microbial activity. Excessive feeding, particularly under cooler conditions, can temporarily suppress bacterial activity, resulting in a pH drop. Prolonged low pH levels can inhibit microbial function, potentially rendering the digester less effective. The findings emphasize the importance of maintaining pH within the optimal range to sustain bacterial activity and methane production. After periods of increased feeding, bacterial adaptation and a rise in temperature above 20°C restored pH levels within 2–4 days, demonstrating the resilience of the microbial community under controlled adjustments.

Previous research on cow manure microbiomes provides valuable context for interpreting the pH variations observed. Meng et al. (2019) demonstrated that microbial succession during composting is strongly influenced by pH and other physicochemical conditions, with Proteobacteria, Bacteroidetes, Firmicutes, and Actinobacteria dominating at different stages [42]. More recent sequencing studies have shown a shift from Firmicutes in fresh manure to increased abundances of Proteobacteria and Bacteroidota after composting, alongside the emergence of taxa such as Myxococcota and Chloroflexota under fluctuating pH conditions [43,44]. These findings highlight the interdependence between microbial community dynamics and pH regulation. Although direct microbial sequencing was not performed in the present study, the observed pH responses align with previously reported trends, suggesting that microbial adaptation played a pivotal role in restoring system stability following environmental disturbances.

### 3.4. Digestive performance assessment

Optimizing biogas production requires continuous evaluation of digester performance. In this study, an automated biogas digester system comprising three independent reactors with individual stirrers was employed, ensuring uniform mixing and effective anaerobic digestion. Daily feeding was conducted following the methodology described in Eq. (2) and the procedure outlined by Rosta (2017) [29]. Initially, each reactor received a constant daily feed of 20 g (equivalent to 0.49 gVS/L) of fresh dairy cow manure for the first seven days. Thereafter, the feeding rate was increased by 20 g every seven days, eventually reaching a maximum of 280 g per day (6.82 gVS/L d) by day 98 of the experiment. This feeding schedule aligns with prior findings [26], where a final feeding rate of 6 kgVS/m³·day over ten days demonstrated effective biogas production. The highest and most stable biogas and methane production was achieved at the maximum feeding rate of 280 g per day, consisting of equal parts fresh manure and water. Average yields under these conditions were 16.2 NL of biogas and 9.2 NL of methane per 280 g of feed. When normalized to the digester’s effective volume, these correspond to biogas and methane yields of 1.3 NL·L ⁻ ¹ and 1.7 NL·L ⁻ ¹, respectively. These results indicate that increasing the feed rate to an optimal threshold enhances both biogas and methane production while maintaining process stability. The findings underscore the importance of precise feeding regimens and demonstrate the potential of well-controlled anaerobic digestion systems to achieve high operational efficiency

### 3.5. Biogas production from digester feeding

**Fig 5:**
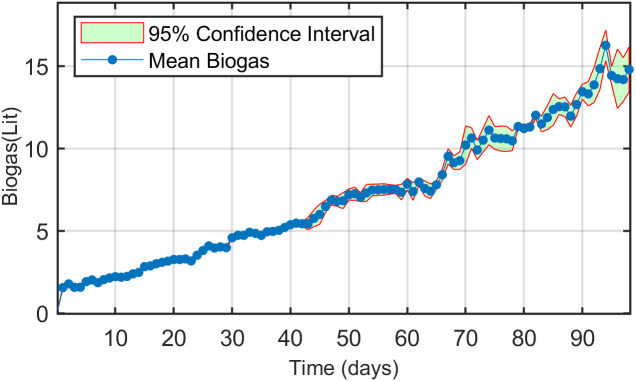
Daily biogas production over the 98-day digestion period based on a feed rate of 35.84 gVS per 5.25 L of inoculum.

Fig 5 illustrates the daily average biogas production over the 98-day experimental period. The feed rate was maintained consistently, resulting in **n**early uniform biogas production across all digesters, indicating minimal experimental variability. Daily feeding positively influenced biogas output within a defined range, starting with 20 g of feed (0.49 gVS/L) and increased by 20 g each week throughout the experiment. Biogas production increased steadily from day 1 to day 55. Between days 56 and 64, production stabilized within a narrow range while the feeding amounts varied between 140 and 160 g (4–5.4 gVS/L). During this period, the digester pH remained stable at 7.25–7.30, and the laboratory temperature ranged from 17°C to 19°C. From day 65 onwards, a marked increase in biogas production was observed, peaking at approximately 9 L/day by day 93. Following this peak, production gradually declined as the feeding rate was reduced. The maximum biogas production occurred on day 94, with a feed amount of 280 g (comprising 280 g fresh cow manure + 280 g water), corresponding to 35.84 gVS per 5.25 L digester volume (6.82 gVS/L). This feeding regimen resulted in a peak biogas yield of 16.25 L. For context, Hidayati et al. (2019) reported that 1 kg of cow manure produced approximately 40 L of biogas, highlighting the consistency of the observed results with previous studies [45].

### 3.6. Methane concentration fluctuations

**Fig 6:**
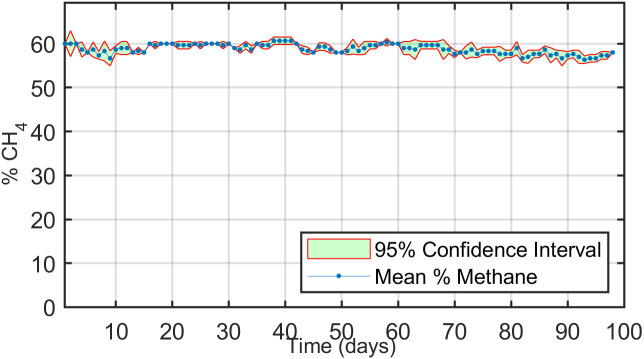
Daily variations in methane concentration (%) during the 98-day anaerobic digestion of cow manure.

During the experiment, methane produced from cow manure fed into the digesters ranged between 56% and 60% (Fig 6). Methane content was measured daily using the MultiTech 545 gas analyzer, and since methane production was similar across all digesters, the average concentration was used for analysis. In the first three weeks, methane production decreased by approximately 3% as 20 g of feed were added weekly. Once the microbial community adapted to the increased nutrient load, methane production recovered by 2%, returning to 58–60%. In the subsequent week, the addition of another 20 g of feed initially caused a drop in methane concentration to 56%. However, with continued feeding, methane levels stabilized again within the 58–60% range. These temporary decreases in methane production at the start of each feeding cycle indicate a brief nutritional shock, where the sudden increase in substrate temporarily exceeded bacterial capacity. This nutrient surge also caused minor shifts in digester pH, tending toward acidification. As the bacterial population grew and activity increased, nutrients were processed efficiently, and pH returned to optimal levels. These results align with previous findings. Salam et al. (2015) reported methane concentrations ranging from 55% to 65% in biogas from continuous digesters, while in our laboratory-scale digesters, methane fluctuated between 56% and 60%, consistent with reported trends [46].

### 3.7. Investigation of daily methane production from digester feeding

**Fig 7:**
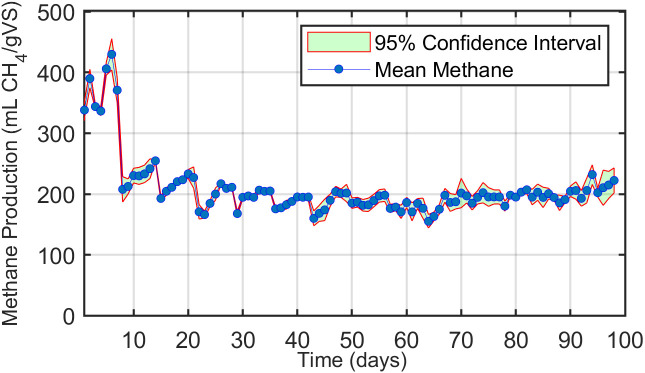
Methane generation per gram of volatile solids during daily feeding with dairy cow manure.

As shown in Fig 7, daily methane yields from continuously fed digesters supplied with dairy cow manure (without additives) were expressed in mL CH₄/gVS. The highest production was recorded during the first three weeks, with a peak of 429 mL CH₄/gVS on day 6. The inoculum used in this study, derived from cow manure (Section 2.2.2), contained bacteria already adapted to the substrate, which likely facilitated their rapid proliferation and activity in the digesters. Despite the initially high bacterial abundance in the inoculum, the addition of a small amount of feed (20 g) stimulated microbial activity and enhanced methane production. This early increase can be attributed to the relatively high bacterial density compared with the available nutrients. However, in the second week, when an additional 20 g of feed was introduced, methane production declined sharply to 207 mL CH₄/gVS on day 8, as the nutrient supply exceeded the metabolic capacity of the bacterial population. Production subsequently began to recover after day 9, reaching 227 mL CH₄/gVS by day 21. During the first and third weeks, nutrient loading ranged from 0.5 to 1.5 gVS/L, respectively. The feeding levels applied in this study were considerably lower than those recommended in previous work, where [29] suggested 5 gVS/L and [26] recommended 6 gVS/L. To gradually reach these higher levels, feed input was increased weekly; however, this adjustment resulted in methane yields fluctuating between 160 and 210 mL CH₄/gVS. The lowest production (155 mL CH₄/gVS) occurred on day 64, one day after the feed load was increased to 4.5 gVS/L. By day 65, methane production had normalized, peaking again on day 94 at 232 mL CH₄/gVS, corresponding to a feed rate of 6.82 gVS/L.

According to Eq. (3.3), the hydraulic retention time (HRT) for a digester with an effective volume of 5.25 L and a daily feed of 280 g was approximately 19 days.

Table 2 presents a comparative evaluation of methane yields under different operational conditions, including total solids (TS), volatile solids (VS), pH, methane concentration (%CH₄), and experimental duration. The methane yields obtained in this study (205–232 mL CH₄/gVS) fall within the ranges previously reported. Nevertheless, variations in feed rates and retention times across studies significantly influenced the observed differences. These results underscore the importance of optimized feeding strategies and controlled operational parameters in maximizing methane production efficiency

**Table 2:** Comparative analysis of methane production and operational parameters across studies.

Maximum methane yield (ml CH_4_/g VS)	TS (%)	VS (%)	pH	CH_4_ (%)	Experimental Duration (Days)	Reference
139.5	19.8	85.7	8.57	53-63	486	[47]
186.9-192.3	23	78-81	7.7-7.8	49-64	250	[48]
205-232	16.18	80.83	7.10-7.40	56-60	98	current study
217	19.41	18.07	8.27	72.3-87.9	90	[49]
68.61	2.64	85.57	–		80	[50]
195_215	22.6	15.5	–	60	70	[51]
170-200	14.46	95.6	7.42	–	43	[52]
240	36	73.2	–	60	45	[53]
204	16.91	10.25	8.1	69	45	[54]
223	15.2	13.8	7.5	50-75	40	[55]
63.42	4.47	3.5	7.47	61-89	40	[56]
201.6	10.8	74.4	7.7	67.2	35	[16]
192-216	14.4	66	7.2		30	[57]
66.5	12.13	10.72	7.25	64	24	[58]
55-69	16.3	13.2	7.5-7.9	32-35	18	[38]
29	21	19	–	65.7	–	[45]
169-270	9.4-22.75	10.25-93.11	5.33-8.30	–	–	[59]

### 3.8. Stability analysis of digestion performance

To evaluate the temporal stability of the anaerobic digestion process, a repeated-measures analysis of variance (RM-ANOVA) was performed on methane production and other key fermentation-related parameters throughout the experimental period. This statistical method accounts for correlations among repeated measurements within the same experimental units, thereby enhancing both the sensitivity and reliability of detecting time-dependent variations. In addition, RM-ANOVA was applied to assess the consistency of four critical process indicators—pH, cumulative biogas production, methane volume, and methane concentration (%CH₄)—under the specified operational conditions. The corresponding statistical outputs, including degrees of freedom (DF), sum of squares (SS), mean square (MS), and p-values, are summarized in Table 3. As shown in Table 3, none of the examined variables demonstrated statistically significant changes over time, with all p-values equal to 0.50. This lack of significance suggests that the observed fluctuations in pH, biogas yield, methane output, and methane content were within a stable operational range rather than representing meaningful variations. Overall, these results confirm that the anaerobic digestion process maintained stability and consistency under the applied conditions, thereby reflecting the robustness of the system as well as the effectiveness of the process control strategies implemented during the experiment

**Table 3:** Summary of repeated measures ANOVA results for key digestion parameters.

Parameter	Degree of freedom	Sum of squares	Mean square	p-value
pH	2	0.42	0.21	0.50
Biogas	2	4.53	2.26	0.50
Methane	2	510.76	255.38	0.50
Methane (%)	2	8.89	4.44	0.50

### 3.9. Analysis of digestate from anaerobic digestion

The essential nutrients—nitrogen (N), phosphorus (P), and potassium (K)—are critical components of soil fertilizers. To evaluate the fertilizer potential of the digestate produced through anaerobic digestion, its nutrient composition was compared with that of both the inoculum used in the digesters and fresh dairy cow manure (Table 1). The results showed a reduction in N and P concentrations in the inoculum, accompanied by an increase in K, relative to fresh cow manure. In contrast, the digestate obtained after anaerobic digestion contained higher levels of N and P compared to both the inoculum and fresh manure. Anaerobically digested manure provides important nutrients such as N, P, K, and Mg, which are essential for plant growth. During digestion, most of the energy from organic matter is converted into methane, while nitrogen largely remains in the form of ammonium (NH₄⁺) within the residue [60]. The digestate is therefore rich in NH₄ ⁺ -N and K, with its fertilizing potential primarily attributable to nitrogen availability [61]. Due to its elevated nitrogen content, digestate can serve as an effective fertilizer for agricultural applications [46].

**Fig 8:**
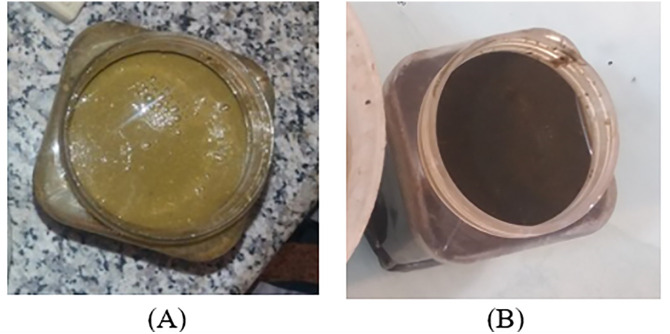
Materials used and generated in the study: (A) Sample prepared for feeding; (B) Digestate obtained from anaerobic digestion.

Prior to digester operation, a digestate sample was collected from each unit. The feed mixture consisted of equal proportions of fresh manure (50%) and water (50%). As shown in Fig 8a, the fresh feed had a high density and a yellowish-brown color. After 24 hours of digestion, the material changed to a darker burnt-brown color as a result of bacterial activity. In addition, the solid fraction decomposed and the strong odor was eliminated. Fresh manure contains organic compounds that are degraded by microorganisms during anaerobic digestion, releasing nitrogen and other nutrients that can partially substitute for cow manure or mineral fertilizers [62]. However, because soil microbial communities rely on organic carbon (C) for growth and activity, continuous application of digested material may gradually reduce soil organic matter reserves [63]. Nevertheless, incorporation of digestate into soil enhances plant growth by supplying readily available nutrients such as N and P and by improving soil biological properties, including microbial biomass and enzyme activities, relative to untreated soils. Notably, although nitrogen from digestate is initially available for rapid plant uptake, it can be lost under cold conditions due to reduced microbial activity, which diminishes its long-term fertilization potential [64].

### 3.10. Material and energy balance assessment

To evaluate the overall efficiency of the anaerobic digestion system, a comprehensive material and energy balance was performed using data from the final and most stable phase of the experiment (days 78–98), during which the digesters were operated at their optimal feeding rate. Each reactor was fed daily with 280 g of substrate, consisting of equal proportions of fresh cow manure and water. Based on the physicochemical characteristics of the manure (16.18% total solids and 80.83% volatile solids on a TS basis; Table 1), the daily input of volatile solids (VS) was calculated as:


$${VS}_{daily}=\text{2}\text{8}\text{0}g\times \text{0}.\text{1}\text{6}\text{1}\text{8}\times \text{0}.\text{8}\text{0}\text{8}\text{3}\approx \text{3}\text{6}.\text{6}gVS/day$$ (7)

Over a 20-day operating period at this feeding rate, the cumulative input of volatile solids was:


$${VS}_{total}=\text{3}\text{6}.\text{6}gVS/day\times \text{2}\text{0}days=\text{7}\text{3}\text{2}gVS$$ (8)

Given the average methane yield of 232 mL CH₄/gVS observed during this period (Fig 7), the total methane production was:


$${CH}_{\text{4}}=\frac{\text{232mL}}{gVS}\times \text{7}\text{3}\text{2}gVS=\text{1}\text{6}\text{9}.\text{8}\text{2}L$$ (9)

Assuming a theoretical methane potential of 350 mL CH₄/gVS for dairy manure-derived substrates [55], the corresponding mass of degraded VS was estimated as:


$${VS}_{degraded}=\;\frac{\text{1}\text{6}\text{9}\text{8}\text{2}\text{4}mL{CH}_{\text{4}}}{\text{3}\text{5}\text{0}mL{CH}_{\text{4}}/gVS}\approx \text{4}\text{8}\text{5}gVS$$ (10)

This corresponds to a VS degradation efficiency of:


$${\text{Efficiency}}_{vs}=\;\frac{\text{4}\text{8}\text{5}gVS}{\text{7}\text{3}\text{2}gVS}\times \text{1}\text{0}\text{0}\approx \text{6}\text{6}\%$$ (11)

The energy output associated with the recovered methane was calculated using the lower heating value (LHV) of methane, approximately 35.8 MJ/m³:


$${\text{Energy}}_{output}=\text{ 169}.\text{82L}{CH}_{\text{4}}\times \text{0}.\text{0}\text{3}\text{5}\text{8}MJ/L\approx \text{6}.\text{0}\text{8}MJ$$ (12)

Consequently, the specific energy yield per unit of volatile solids fed was:


$${\text{Energy}}_{yield}=\;\frac{\text{6}.\text{0}\text{8}MJ}{\text{0}.\text{7}\text{3}\text{2}kgVS}\approx \text{8}.\text{3}\text{0}MJ/kgVS$$ (13)

These findings indicate a high energy conversion efficiency under mesophilic conditions and controlled feeding, achieving over 66% VS degradation and an energy yield of approximately 8.3 MJ/kgVS. The results highlight the robustness of the digestion process and the effectiveness of the feeding strategy in maximizing methane recovery and overall energy output

## Conclusion

This study examined the effect of incrementally increasing the feeding rate of cow manure on biogas production under mesophilic conditions, with the aim of identifying an optimal feeding strategy to enhance process performance. Feeding was increased weekly in steps of 20 g, reaching a final input of 280 g per day per 5.25 L of inoculum (6.82 gVS/L) by the end of the 98-day experiment.

Biogas production increased progressively during the early phase (days 1–55), followed by a stabilization period (days 56–64). Methane production then rose again and peaked on day 94, with yields closely linked to higher feeding levels. Although statistical analysis (RM-ANOVA, p = 0.50) showed no significant differences among treatments, the observed patterns suggest that incremental feeding contributed to meaningful improvements in both process stability and cumulative methane yield over time.

Digestate analysis revealed higher concentrations of nitrogen (N), phosphorus (P), and potassium (K) compared with the initial inoculum and fresh manure, underscoring its value as a nutrient-rich organic fertilizer. Despite short-term fluctuations, the digestion process eventually stabilized, demonstrating the adaptability of the microbial community to the feeding regime. This stability highlights the practical importance of gradual feeding adjustments in maintaining reliable digester operation.

In conclusion, although the improvements observed were not statistically significant, the findings emphasize the practical relevance of controlled feeding for optimizing methane yield, ensuring process stability, and improving digestate quality. These results provide useful guidance for managing anaerobic digestion systems in both research and applied contexts. Beyond energy recovery, the nutrient recycling potential of digestate further illustrates the contribution of anaerobic digestion to sustainable agriculture and the circular bioeconomy. Future research should focus on the scalability of incremental feeding strategies and the long-term agronomic benefits of digestate application.

## Supporting information

S1 Data (RAR)

## References

[1] Ma J, Shang Y, Liang Z. National central cities, technological innovation, and economic growth. Finance Research Letters. 2024;67(PB):105890.

[2] Erixno O, Ramadhani F, Abd Rahim N, Rivai A. Realistic operation and strategic energy, economic and environmental analyses of a hybrid renewable energy based - micro combined heat and power. Solar Energy. 2024;272:112467.

[3] Rahman A, Murad SW, Mohsin AK, Wang X. Does renewable energy proactively contribute to mitigating carbon emissions in major fossil fuels consuming countries?. Journal of Cleaner Production. 2024;452:142113.

[4] Ersoy AE, Ugurlu A. Bioenergy’s role in achieving a low-carbon electricity future: A case of Türkiye. Applied Energy. 2024;372:123799.

[5] Negro V, Noussan M, Chiaramonti D. Alternative options for biogas-to-energy: A comparison of electricity and biomethane generation based on the real operation of a production site. Applied Energy. 2025;377:124687.

[6] Subbarao PMV, D’ Silva TC, Adlak K, Kumar S, Chandra R, Vijay VK. Anaerobic digestion as a sustainable technology for efficiently utilizing biomass in the context of carbon neutrality and circular economy. Environ Res. 2023;234:116286. doi: 10.1016/j.envres.2023.116286 37263473

[7] Zhang H, et al. Anaerobic digestion based waste-to-energy technologies can halve the climate impact of China’s fast-growing food waste by 2040. Journal of Cleaner Production. 2020;277:123490.

[8] Prasad S. Review on biofuel production: Sustainable development scenario, environment, and climate change perspectives − A sustainable approach. Journal of Environmental Chemical Engineering. 2024;12(2):111996.

[9] Alengebawy A. Anaerobic digestion of agricultural waste for biogas production and sustainable bioenergy recovery: a review. Environmental Chemistry Letters. 2024;22(6):2641–68.

[10] Alharbi RM. Anaerobic co-digestion of cow manure and microalgae to increase biogas production: a sustainable bioenergy source. Journal of King Saud University-Science. 2024;36(9):103380.

[11] Font-Palma C. Methods for the Treatment of Cattle Manure—A Review. C. 2019;5(2):27. doi: 10.3390/c5020027

[12] Hangri S, Derbal K, Policastro G, Panico A, Contestabile P, Pontoni L, et al. Combining pretreatments and co-fermentation as successful approach to improve biohydrogen production from dairy cow manure. Environ Res. 2024;246:118118. doi: 10.1016/j.envres.2024.118118 38199469

[13] Kusmiyati K, Wijaya DK, Hartono BR, Shidik GF, Fudholi A. Harnessing the power of cow dung: Exploring the environmental, energy, and economic potential of biogas production in Indonesia. Results in Engineering. 2023;20:101431.

[14] Jomnonkhaow U, Uwineza C, Mahboubi A, Wainaina S, Reungsang A, Taherzadeh MJ. Membrane bioreactor-assisted volatile fatty acids production and in situ recovery from cow manure. Bioresour Technol. 2021;321:124456. doi: 10.1016/j.biortech.2020.124456 33276207

[15] Atienza-Martínez M. Pyrolysis of dairy cattle manure: evolution of char characteristics. Journal of Analytical and Applied Pyrolysis. 2020;145:104724.

[16] Hilmi N, Zakarya IA, Gunny AA, Izhar TT, Zaaba SK, Samah MF, et al. Co-digestion of food waste with cow dung by anaerobic digestion for biogas production. In: IOP Conference Series: Earth and Environmental Science, 2023.

[17] Kasulla S, Malik SJ, Bhatta AD, Kathpal G, Yadav A. et al. Process monitoring of biogas projects. 2024.

[18] Chojnacka K, Moustakas K. Anaerobic digestate management for carbon neutrality and fertilizer use: A review of current practices and future opportunities. Biomass and Bioenergy. 2024;180:106991.

[19] Tiong YW, Sharma P, Xu S, Bu J, An S, Foo JBL, et al. Enhancing sustainable crop cultivation: The impact of renewable soil amendments and digestate fertilizer on crop growth and nutrient composition. Environ Pollut. 2024;342:123132. doi: 10.1016/j.envpol.2023.123132 38081377

[20] Wang K, Yun S, Xing T, Li B, Abbas Y, Liu X. Binary and ternary trace elements to enhance anaerobic digestion of cattle manure: Focusing on kinetic models for biogas production and digestate utilization. Bioresour Technol. 2021;323:124571. doi: 10.1016/j.biortech.2020.124571 33388599

[21] Zhang X, Jiao P, Zhang M, Wu P, Zhang Y, Wang Y, et al. Impacts of organic loading rate and hydraulic retention time on organics degradation, interspecies interactions and functional traits in thermophilic anaerobic co-digestion of food waste and sewage sludge. Bioresour Technol. 2023;370:128578. doi: 10.1016/j.biortech.2023.128578 36610483

[22] Greses S, Jimenez J, González-Fernández C, Steyer J-P. Modelling of anaerobic digestion of microalgae biomass: Effect of overloading perturbation. Bioresour Technol. 2024;399:130625. doi: 10.1016/j.biortech.2024.130625 38518882

[23] Sun S, Wang X, Cheng S, Lei Y, Sun W, Wang K, et al. A review of volatile fatty acids production from organic wastes: Intensification techniques and separation methods. J Environ Manage. 2024;360:121062. doi: 10.1016/j.jenvman.2024.121062 38735068

[24] Wang S, Li D, Zhang K, Ma Y, Liu F, Li Z, et al. Effects of initial volatile fatty acid concentrations on process characteristics, microbial communities, and metabolic pathways on solid-state anaerobic digestion. Bioresour Technol. 2023;369:128461. doi: 10.1016/j.biortech.2022.128461 36503086

[25] Zhao W, Jeanne Huang J, Hua B, Huang Z, Droste RL, Chen L, et al. A new strategy to recover from volatile fatty acid inhibition in anaerobic digestion by photosynthetic bacteria. Bioresour Technol. 2020;311:123501. doi: 10.1016/j.biortech.2020.123501 32416492

[26] Chala B, Oechsner H, Fritz T, Latif S, Müller J. Increasing the loading rate of continuous stirred tank reactor for coffee husk and pulp: Effect of trace elements supplement. Eng Life Sci. 2018;18(8):551–61. doi: 10.1002/elsc.201700168 32624935 PMC6999297

[27] Luo L, Chen J. Integrating anaerobic digestion with ground source heat pump system for co-generation of heating, cooling and biogas: An economic and environmental analysis and process optimization. Energy. 2025;322:135437.

[28] Association, A.P.H. and A.P.H. Association, Standard methods for the Examination of Water and Wastewater, APHA. American Water Works Association and Water Environment Federation, 21st ed.; Washington, DC, USA: American Public Health Association, 2005.

[29] Rosato MA. Managing biogas plants: A practical guide. CRC Press. 2017.

[30] Neubauer L, Krümpel J, Khan MT, Lemmer A. Predicting anaerobic digestion stability in load-flexible operation using gas phase indicators and classification algorithms. Bioresour Technol. 2025;429:132508. doi: 10.1016/j.biortech.2025.132508 40209911

[31] Liu C, Guo T, Chen Y, Meng Q, Zhu C, Huang H. Physicochemical characteristics of stored cattle manure affect methane emissions by inducing divergence of methanogens that have different interactions with bacteria. Agriculture, Ecosystems & Environment. 2018;253:38–47.

[32] Wang H, Aguirre-Villegas HA, Larson RA, Alkan-Ozkaynak A. Physical Properties of Dairy Manure Pre- and Post-Anaerobic Digestion. Applied Sciences. 2019;9(13):2703. doi: 10.3390/app9132703

[33] Miah MR, Rahman AK, Akanda MR, Pulak A, Rouf MA. Production of biogas from poultry litter mixed with the co-substrate cow dung. Journal of Taibah University for Science. 2016;10(4):497–504.

[34] Chen G, Zheng Z, Yang S, Fang C, Zou X, Zhang J. Improving conversion of Spartina alterniflora into biogas by co-digestion with cow feces. Fuel processing technology. 2010;91(11):1416–21.

[35] Ren Y, Li Y, He Z, Qin Y, Sakamaki T, Li YY. et al. System stability associated with different lipid contents during mesophilic anaerobic digestion of lipid-rich food waste. Journal of Cleaner Production. 2024;443:141171.

[36] Yellezuome D. Mitigation of ammonia inhibition in anaerobic digestion of nitrogen-rich substrates for biogas production by ammonia stripping: A review. Renewable and Sustainable Energy Reviews. 2022;157:112043.

[37] Ikram M, Mehran M, Minhas A, ur Rehman H, Bakash MZ, Khan MW, et al. C:N Ratio and Its Importance in Developing Effective Bioenergy Crops. In: Singhal RK. Forage Crops in the Bioenergy Revolution: From Fields to Fuel. Singapore: Springer Nature Singapore. 2025. 259–76.

[38] Zhang C, Xiao G, Peng L, Su H, Tan T. The anaerobic co-digestion of food waste and cattle manure. Bioresour Technol. 2013;129:170–6. doi: 10.1016/j.biortech.2012.10.138 23246757

[39] Abdelsalam E, Samer M, Attia YA, Abdel-Hadi MA, Hassan HE, et al. Comparison of nanoparticles effects on biogas and methane production from anaerobic digestion of cattle dung slurry. Renewable Energy. 2016;87:592–8.

[40] Prasanna Kumar DJ, Mishra RK, Chinnam S, Binnal P, Dwivedi N. A comprehensive study on anaerobic digestion of organic solid waste: A review on configurations, operating parameters, techno-economic analysis and current trends. Biotechnol Notes. 2024;5:33–49. doi: 10.1016/j.biotno.2024.02.001 39660169 PMC11630644

[41] Atasoy M, Cetecioglu Z. The effects of pH on the production of volatile fatty acids and microbial dynamics in long-term reactor operation. J Environ Manage. 2022;319:115700. doi: 10.1016/j.jenvman.2022.115700 35982552

[42] Meng Q, Yang W, Men M, Bello A, Xu X, Xu B, et al. Microbial Community Succession and Response to Environmental Variables During Cow Manure and Corn Straw Composting. Front Microbiol. 2019;10:529. doi: 10.3389/fmicb.2019.00529 30936861 PMC6431636

[43] Zalewska M, Błażejewska A, Szadziul M, Ciuchciński K, Popowska M. Effect of composting and storage on the microbiome and resistome of cattle manure from a commercial dairy farm in Poland. Environ Sci Pollut Res Int. 2024;31(21):30819–35. doi: 10.1007/s11356-024-33276-z 38616224 PMC11096248

[44] Tharek M, Mat Amin N. Bacterial community structure of fresh and composted cattle manure revealed through 16S rRNA gene amplicon sequencing. Microbiol Resour Announc. 2025;14(4):e0109224. doi: 10.1128/mra.01092-24 40130926 PMC11984130

[45] Hidayati S, et al. Technical and technology aspect assessment of biogas agroindustry from cow manure: case study on cattle livestock industry in South Lampung District. In: IOP Conference Series: Earth and Environmental Science, 2019.

[46] Salam B, Biswas S, Rabbi MS. Biogas from mesophilic anaerobic digestion of cow dung using silica gel as catalyst. Procedia Engineering. 2015;105:652–7.

[47] Chiumenti A, da Borso F, Limina S. Dry anaerobic digestion of cow manure and agricultural products in a full-scale plant: Efficiency and comparison with wet fermentation. Waste Manag. 2018;71:704–10. doi: 10.1016/j.wasman.2017.03.046 28389052

[48] Ahlberg-Eliasson K, Westerholm M, Isaksson S, Schnürer A. et al. Anaerobic digestion of animal manure and influence of organic loading rate and temperature on process performance, microbiology, and methane emission from digestates. Frontiers in Energy Research. 2021;9:740314.

[49] Li K, Liu R, Sun C. Comparison of anaerobic digestion characteristics and kinetics of four livestock manures with different substrate concentrations. Bioresour Technol. 2015;198:133–40. doi: 10.1016/j.biortech.2015.08.151 26386415

[50] Budiyono B. Increasing biogas production rate from cattle manure using rumen fluid as inoculums. International Journal of Science and Engineering. 2014;6(1):31–8.

[51] Abdallah M, Shanableh A, Adghim M, Ghenai C, Saad S. Biogas production from different types of cow manure. In: 2018 Advances in Science and Engineering Technology International Conferences (ASET), 2018.

[52] Triolo JM, Ward AJ, Pedersen L, Sommer SG. Characteristics of animal slurry as a key biomass for biogas production in Denmark. Biomass Now-Sustainable Growth and Use. Intech. 2013. 307–26.

[53] Osman GA, Elhasan HE, Hassan AB. Effect of cow rumen fluid concentration on biogas production from goat manure. Sudan J Agric Sci. 2015;2:1–7.

[54] Kafle GK, Chen L. Comparison on batch anaerobic digestion of five different livestock manures and prediction of biochemical methane potential (BMP) using different statistical models. Waste Manag. 2016;48:492–502. doi: 10.1016/j.wasman.2015.10.021 26531046

[55] Khairuddin N. High solid anaerobic co-digestion of household organic waste with cow manure. Procedia Environmental Sciences. 2015;30:174–9.

[56] Aili Hamzah AF, Hamzah MH, Che Man H, Jamali NS, Siajam SI, Ismail MH. Effect of organic loading on anaerobic digestion of cow dung: Methane production and kinetic study. Heliyon. 2023;9(6):e16791. doi: 10.1016/j.heliyon.2023.e16791 37303543 PMC10250787

[57] Almomani F, Shawaqfah M, Bhosale RR, Kumar A, Khraisheh MA. Intermediate ozonation to enhance biogas production in batch and continuous systems using animal dung and agricultural waste. International Biodeterioration & Biodegradation. 2017;119:176–87.

[58] Achinas S, Li Y, Achinas V, Euverink GJ. Influence of sheep manure addition on biogas potential and methanogenic communities during cow dung digestion under mesophilic conditions. Sustainable Environment Research. 2018;28(5):240–6.

[59] Caruso MC. Recent updates on the use of agro-food waste for biogas production. Applied Sciences. 2019;9(6):1217.

[60] Lehto J, Järvelä E. Valorisation of anaerobic digestate to nutrients and humic substances. Waste Manag. 2025;192:39–46. doi: 10.1016/j.wasman.2024.11.033 39580949

[61] Rizzioli F, Cirilli M, Frison N, Bolzonella D, Battista F. et al. Nutrient recovery from anaerobic digestate by different combination of pressure driven membranes. Journal of Cleaner Production. 2025;494:144958.

[62] Essalhi F, Naourani A, Essadek A, Bengueddour R. Agronomic and energy value of digestate from anaerobic digestion of trout byproducts: Contribution to the autonomy of freshwater farms in Morocco. Results in Engineering. 2024;23:102508.

[63] Vautrin F, Piveteau P, Cannavacciuolo M, Barré P, Chauvin C, Villenave C, et al. The short-term response of soil microbial communities to digestate application depends on the characteristics of the digestate and soil type. Applied Soil Ecology. 2024;193:105105.

[64] Cucina M, Massaccesi L, Garfí M, Saponaro V, Muñoz Muñoz A, Escalante H, et al. Application of digestate from low-tech digesters for degraded soil restoration: Effects on soil fertility and carbon sequestration. Sci Total Environ. 2025;967:178854. doi: 10.1016/j.scitotenv.2025.178854 39954479

